# Impact of an educational intervention on patient safety culture among gynecology-obstetrics’ healthcare professionals

**DOI:** 10.1186/s12913-024-11152-3

**Published:** 2024-06-05

**Authors:** Latifa Lassoued, Ines Gharssallah, Mohamed Ayoub Tlili, Jihene Sahli, Mouna Kouira, Skender Abid, Anouar Chaieb, Hedi Khairi

**Affiliations:** 1 Université de Sousse, Faculté de Médecine de Sousse, Sousse, LR12ES03, 4000 Tunisie; 2 Service de Gynécologie Obstétrique CHU Farhat Hached, Sousse, 4000 Tunisie; 3 Department of Nursing Administration, College of Nursing, University of Hail, Hail, Saudi Arabia

**Keywords:** Patient safety, Patient safety culture, Quality of care, Gynecology-Obstetrics

## Abstract

**Background:**

In recent years, patient safety has begun to receive particular attention and has become a priority all over the world. Patient Safety Culture (PSC) is widely recognized as a key tenet that must be improved in order to enhance patient safety and prevent adverse events. However, in gynecology and obstetrics, despite the criticality of the environment, few studies have focused on improving PSC in these units. This study aimed at assessing the effectiveness of an educational program to improve PSC among health professionals working in the obstetric unit of a Tunisian university hospital.

**Methods:**

We conducted a quasi-experimental study in the obstetric unit of a university hospital in Sousse (Tunisia). All the obstetric unit’s professionals were invited to take part in the study (*n* = 95). The intervention consisted of an educational intervention with workshops and self-learning documents on patient safety and quality of care. The study instrument was the French validated version of the Hospital Survey on Patient Safety Culture. Normality of the data was checked using Kolmogorov-Smirnov test. The comparison of dimensions’ scores before and after the intervention was carried out by the chi2 test. The significance level was set at 0.05.

**Results:**

In total, 73 participants gave survey feedback in pre-test and 68 in post-test (response rates of 76.8% and 71.6, respectively). Eight dimensions improved significantly between pre- and post-tests. These dimensions were D2 “Frequency of adverse events reported” (from 30.1 to 65.6%, *p* < 0.001), D3 “Supervisor/Manager expectations and actions promoting patient safety” (from 38.0 to 76.8%, *p* < 0.001), D4 “Continuous improvement and organizational learning” (from 37.5 to 41.0%, *p* < 0.01), D5 “Teamwork within units” (from 58.2 to 79.7%, *p* < 0.01), D6 “Communication openness” (from 40.6 to 70.6%, *p* < 0.001), and D7 “Non-punitive response to error” (from 21.1 to 42.7%, *p* < 0.01), D9 “Management support for patient safety” (from 26.4 to 72.8%, *p* < 0.001), and D10 “Teamwork across units” (from 31.4 to 76.2%, *p* < 0.001).

**Conclusions:**

Educational intervention, including workshops and self-learning as pedagogical tools can improve PSC. The sustainability of the improvements made depends on the collaboration of all personnel to create and promote a culture of safety. Staff commitment at all levels remains the cornerstone of any continuous improvement in the area of patient safety.

## Introduction

In recent years, patient safety has begun to receive particular attention and has become a priority all over the world due to the persistent high and rising incidence of adverse events (AEs), their impact, and the availability of efficient and practical preventative measures [1–3]. The incidence of AEs in hospitalized patients ranges from 3 to 17% and up to 50% of AEs are deemed preventable [4].

Among the numerous healthcare settings, obstetrics units are areas of care that particularly account for a significant amount of complaints [4, 5]. They present a significant risk of morbidity and mortality for both mothers and fetus, requiring an effective safety environment to avoid negative outcomes [4, 5]. Providing obstetric care involves several interrelated factors making it at a higher risk of errors than other specialties [6]. These include the diversity of professions, activities and equipment, lack of professional communication, uncertainties, fear of legal action, and a low tolerance for errors. Furthermore, the heavy workload, dangers associated with emergency calls, and the fact that medical personnel is constantly responsible for both the mother and the fetus/newborn, make clinical activities in these units very challenging [6].

In a Swedish research, 12.2% of adverse occurrences were found, and 73.7% of those were deemed to be avoidable [5]. In a recent international meta-analysis the incidence of AEs in gynecological hospital admissions was 10.8%, of which 52.5% could have been avoided and 1.2% were associated with death [7].

In Tunisia, a study revealed that the incidence of AEs in a university hospital was of 12.4%, with hospital acquired infection and unplanned readmission were the most common AEs [8]. Another study reported that the overall incidence of AEs in surgery departments was 18.1%, of which 62% were considered preventable [9]. A prospective study carried out among all patients hospitalized at a Tunisian university hospital in fourteen departments, including in gynecology-obstetrics highlighted that, in total, 162 serious AEs were identified and they resulted in death among 9.2% of patients, life-threatening prognosis for 26% of patients and extended length of stay among 61. 7% of them [10].

Given the magnitude of the problem, Patient Safety Culture (PSC) is widely recognized as a key tenet that must be improved in order to improve patient safety and prevent AEs ; a developed PSC is linked to improved patient outcomes in terms of medication errors, patients’ falls, and pressure ulcers [11]. Aware of the importance of improving PSC, several types of action intended to develop its level have been tested [12, 13]. However, in gynecology and obstetrics, despite the criticality of the environment, few studies have focused on improving PSC in these units [14, 15].

In Tunisia, several studies have delved into PSC within various healthcare settings, consistently indicating a suboptimal level of PSC that warrants enhancement. Notably, these studies were predominantly cross-sectional, non-interventional ones and none of them have been conducted in gynecology-obstetrics.

Highlighting these existing challenges underscores the pressing need for comprehensive interventions and targeted research endeavors tailored to this unique healthcare context with both facility-level improvements and broader policy development initiatives. This study aimed to assess the effectiveness of an educational intervention designed to improve PSC among health professionals working in Gynecology and Obstetrics unit of a Tunisian university hospital.

## Methods

### Study design, settings and participants

We conducted a quasi-experimental study (before – after study). The baseline assessment of PSC (M1) was conducted in June 2021. Then, the intervention, that consisted of a training program on patient safety and risk management, was conducted in July 2021, and followed by a post-intervention assessment (M2) 6 months later (January 2022).

The target population consisted of all healthcare professionals (*n* = 95) working in the obstetrics and gynecology department of Farhat Hached university hospital of Sousse, Tunisia (physicians, midwives, and nurses).

### Study instrument and data collection

The pre- and post-intervention assessments of PSC (M1 and M2) were conducted using the French validated version of the Hospital Survey On Patient Safety Culture (HSOPSC) questionnaire [16].

Participants were provided access to the online questionnaire through a secure platform. The online form allowed for efficient data collection and minimized logistical challenges associated with paper-based forms, such as data entry errors and storage constraints. Responses were automatically collected and stored securely within the platform.

The HSOPSC is composed of 40 items grouped in ten dimensions which are: Overall perceptions of safety (D1), Frequency of events reported (D2), Supervisor/Manager expectations and actions promoting patient safety (D3), Organizational learning and continuous improvement (D4), Teamwork within units (D5), Communication openness (D6), Non-punitive response to error (D7), Staffing (D8), Management support for patient safety (D9), and Teamwork across units (D10). Two additional items explore professionals’ perception of patient safety level and the frequency of AEs reported in the previous 12 months. A 5-point Likert scale with agreement (from “strongly disagree” to “strongly agree”) or frequency (from “never” to “always”) is used to capture participants’ perceptions. Internal consistency of the study instrument was checked; Cronbach’s alpha was of 0.81 for the whole questionnaire and rang ed between 0.72 and 0.87 for the dimensions.

### Intervention

Following completion of the questionnaire (M1), the participants engaged in an active training program that included “workshops” and self-learning. The learning objectives of the training program were to: 1- Situate patient safety as a dimension of quality, 2- Recognize the nature of errors, 3- Learn about risk management tools and methods, 4- Describe the process for reporting AEs associated with care, 5- Analyze AEs associated with care using a systematic approach and 6- Identify the basis for developing a culture of safety. A written educational material addressing the aforementioned objectives was created for self-learning and given to all professionals.

The health professionals who took part in our study then benefitted from a training program that was conducted in July 2021 at the Obstetrics and Gynecology department of Farhat Hached University Hospital of Sousse and at the Unit of Continuous Professional Development in the Medical Education Center of the Faculty of Medicine Ibn El Jazzar of Sousse.

The training program consisted of an introductive session on patient safety and quality of care by the department head-chief, followed by three workshops:

#### Workshop 1 “Room of errors”

In the room of a fully reconstructed prenatal patient, the facilitators introduce a limited number of errors (between 8 and 10). The healthcare professional must then identify the errors when passing through the “error room”. Afterwards, the facilitator presents the various errors noted and explains good practices relating to.

#### Workshop 2 “AEs reporting system”

After a brief introduction on the principles and interest of an AE reporting system, participants described the process of reporting an AE, filled in the report form with a recent AE, and described the committee’s process for handling and analyzing the AE. Each group presented the work in plenary. The other groups can intervene and a discussion was opened.

The facilitator reviewed the essential steps for reporting and analyzing AEs with concrete examples. A particular focus was posed on the systemic nature of AEs, the climate of tolerance and blame-free environment that should be established, and the importance of feedback.

#### Workshop 3 “Analyzing AEs: focus on ALARM method”

The participants did an individual reading of an AE case study. In groups, the participants analyzed the AE according to the ALARM method (that stands for Association of Litigation and Risk Management), which states that participants: 1-Reconstitute the chronology of the facts that led to the event, 2-Identify in this description the faults of care, 3-For each care defect identified, conduct an analysis of the factors that contributed to its occurrence, and 4- Propose corrective actions, and how to evaluate and monitor them using indicators.

Afterwards, a plenary analysis was carried out and finally, a synthesis by the facilitator is presented.

All healthcare professionals received recordings of the face-to-face workshops that were conducted.

The various workshops carried out focused particularly on several barriers to reporting reported in literature and on the systemic approach to the occurrence of AEs and insisted on the fact that the main objective of reporting and identifying the root cause of the AE without pointing the finger at the person who committed it. A specific attention was given to feedback and its importance where we insisted that is extremely crucial that the investigation and the analysis, as well as the proposed solutions should be communicated to the team members so that the errors are not reproduced by another person and can learn from it.

Additionally, during the intervention, concrete examples of improvement actions that were made following AEs occurrence were presented, including the training sessions and the AEs prevention policies in response to reports received.

The post-intervention assessment of PSC (M2) was conducted six months later, on January 2022. This decision was motivated by the fact that some PSC domains require time to show results. It is important to mention that no other interventions were performed during this follow-up period and that the participants did not take part in any other seminars or training.

### Data analysis

Data analysis was conducted using SPSS 26 software (IBM, SPSS Inc., Chicago, IL, USA) and EpiInfo 6.04d, with descriptive analysis displaying the frequencies, percentages, means and standard deviations.

The score of each dimension was calculated based on the Average Positive Response (APR) rate for each item. As items were phrased in both positive and negative ways, items with negative wording were reversely coded, so that a higher score reflects a more developed PSC. A dimension was considered “developed” if it had a score of 75% or above, whereas a score of 50% or less indicates that it is “in need of improvement” [17].

Normality of the data was checked using Kolmogorov-Smirnov test. The comparison of dimensions’ scores (APRs) before and after the intervention as well as differences between the different subgroups of the items “patient safety level” and “number of AEs reported” was carried out by the chi2 test. The significance level was set at 0.05.

### Ethical considerations

This study was approved by the Ethics Committee of the Faculty of Medicine of Sousse. Authorization from the Obstetrics and Gynecology department head-chief was obtained before starting the study. Questionnaires were distributed after the participants gave their consent. Participants were informed of the nature and objective of the study as well as having the right to decline participation or withdraw the study at any time and for any reason. Measures to safeguard participant privacy were taken by anonymizing responses and restricting access to authorized research team only.

## Results

In total, during baseline assessment,73 professionals accepted to take part in the study with a participation rate of 76.8%. With 68 participants in the post-intervention measurement, the participation rate was of 71.6%.

### Characteristics of the participants

Most of the participants were physicians in pre-test (52.0%, *n* = 38) and they represented 44.1% in the post-test (*n* = 30). Table 1 summarizes the participants’ socio-demographic and professional data before and after the intervention.

**Table 1:** Participants’ characteristics

Characteristics	Pre-test (*n* = 73) *n* (%)	Post-test (*n* = 68) *n* (%)
**Professional grade**		
Physician	38 (52.0)	30 (44.10)
Midwives	31(42.50)	32(47.10)
Nurses	4 (5.50)	6(8.80)
**Professional experience**		
< 3 years	13 (17.80)	17 (25.00)
≥ 3 years	60 (82.20)	51 (75.00)
**Work experience in the department**		
< 3 years	25(34.20)	24(35.30)
≥ 3 years	48(65.80)	44(64.70)
**Participation in risk management committees**		
Yes	66 (90.40)	50 (73.50)
No	7 (9.60)	18 (26.50)

### Impact of the intervention on patient safety level and reporting AEs

In terms of patient safety level and number of AEs reported, there was no significant change in participants’ responses (Table 2).

**Table 2:** Impact of the intervention on patient safety level and number of AEs reported

	Pre-test (*n* = 73) *n* (%)	Post-test (*n* = 68) *n* (%)	*P* value (M1-M2)
**Patient safety level**			
Excellent/ very good	13 (17.8)	20 (29.4)	0.458
Acceptable	40 (54.8)	43 (63.2)	0.439
Poor/ failing	20 (27.4)	5 (7.4)	0.354
**Reporting AEs**			
None	62 (87.7)	61 (89.7)	0.727
At least once	9 (12.3)	7 (10.3)	0.903

### Impact of the intervention on PSC dimensions

During pre-test, the least developed dimensions were D8 “Staffing”, D7 “Non-punitive response to error”, and D9 “Management support for patient safety”, with a scores of 18.7%, 21.1%, and 26.4%, respectively. The most developed dimension was D5 “Teamwork within units” with a score of 58.2%.

After the intervention, eight dimensions improved significantly. Changes in PSC between M1 and M2 are presented in Table 3. The best improvement were noted in D9 “Management support for patient safety” (from 26.4 to 72.8%, *p* < 0.001), D10 “Teamwork across units” (from 31.4 to 76.2%, *p* < 0.001), D3 “Supervisor/Manager expectations and actions promoting patient safety” (from 38.0 to 76.8%, *p* < 0.001), and D2 “Frequency of adverse events reported” (from 30.1 to 65.6%, *p* < 0.001),. Items’ APR related to each of the ten dimensions are presented in Table 4.

**Table 3:** Impact of the intervention on PSC dimensions

Dimension	Pre-test Score (%)	Post-test Score (%)	*p* value
D1	40.10	55.90	0.0614
**D2**	**30.10**	**65.60**	**< 0.001**
**D3**	**38.00**	**76.80**	**< 0.001**
**D4**	**37.50**	**61.00**	**< 0.01**
**D5**	**58.20**	**79.70**	**< 0.01**
**D6**	**40.60**	**70.60**	**< 0.001**
**D7**	**21.10**	**42.70**	**< 0.01**
D8	18.70	21.10	0.7221
**D9**	**26.40**	**72.80**	**< 0.001**
**D10**	**31.40**	**76.20**	**< 0.001**

D1: Overall perceptions of safety, D2: Frequency of events reported, D3: Supervisor/Manager expectations and actions promoting patient safety, D4: Organizational learning and continuous improvement, D5:Teamwork within units, D6: Communication openness, D7: Non−punitive response to error, D8: Staffing, D9: Management support for patient safety, D10: Teamwork across units

**Table 4:** Scores and items’ APR of the 10 dimensions of PSC before and after intervention

	Pre-test	Post-test
PSC dimensions and items	APR (%)	APR (%)
**D1: Overall perceptions of safety**	40.1	55.9
“Patient safety is never sacrificed to get more work done”	54.8	75.0
“Our procedures and systems are good at preventing errors from happening”	32.9	70.6
“It is just by chance that more serious mistakes do not happen around here”	54.8	72.0
“We have patient safety problems in this facility”	17.8	5.9
**D2: Frequency of events reported**	**30.1**	**65.6**
“When a mistake is made, but is caught and corrected before affecting the patient, it is reported”	34.3	73.5
“When a mistake is made, but has no potential to harm the patient, it is reported”	24.6	67.7
“When a mistake is made that could harm the patient, but does not, it is reported”	31.5	61.7
**D3: Supervisor/Manager expectations and actions promoting patient safety**	**38.0**	**76.8**
“Manager says a good word when he/she sees a job done according to established patient safety procedures”	32.9	75.0
“Manager seriously considers staff suggestions for improving patient safety”	35.6	76.5
“Whenever pressure builds up, my manager wants us to work faster, even if it means taking shortcuts”	38.4	75.0
“My manager overlooks patient safety problems that happen over and over”	45.2	80.9
**D4: Organizational learning and continuous improvement**	**37.5**	**61.0**
“We are actively doing things to improve patient safety”	42.5	47.1
“Mistakes have led to positive changes here”	41.1	51.4
“After we make changes to improve patient safety, we evaluate their effectiveness”	37.0	27.9
“We are given feedback about changes put into place based on event reports”	01.2	83.8
“We are informed about errors that happen in the facility”	43.8	73.6
“In this facility, we discuss ways to prevent errors from happening again”	41.1	82.4
**D5: Teamwork within units**	**58.2**	**79.7**
“People support one another in this facility”	54.8	83.8
“When a lot of work needs to be done quickly, we work together as a team to get the work done”	74.0	75.0
“In facility, people treat each other with respect”	46.6	76.4
“When one area in this unit gets really busy, others help out”	57.5	83.8
**D6: Communication openness**	**40.6**	**70.6**
“Staff will freely speak up if they see something that may negatively affect patient care”	47.9	73.6
“Staff feel free to question the decisions or actions of those with more authority”	23.3	61.7
“Staff are afraid to ask questions when something does not seem right”	50.7	76.5
**D7: Non-punitive response to error**	**21.1**	**42.7**
“Staff feel like their mistakes are held against them”	21.9	7.4
“When an event is reported, it feels like the person is being written up, not the problem”	27.4	82.4
“Staff worry that mistakes they make are kept in their personnel file”	13.9	38.3
**D8: Staffing**	**18.7**	**21.1**
“We have enough staff to handle the workload”	24.6	42.7
“Staff in this facility work longer hours than is best for patient care”	11.0	04.4
“We work in ‘crisis mode’ trying to do too much, too quickly”	20.5	16.2
**D9: Management support for patient safety**	**26.4**	**72.8**
“Management provides a work climate that promotes patient safety”	21.9	69.1
“The actions of management show that patient safety is a top priority”	20.6	72.0
“Management seems interested in patient safety only after an adverse event happens”	19.2	75.0
“Units work well together to provide the best care for patients”	43.9	75.0
**D10: Teamwork across units**	**31.8**	**76.2**
“There is good cooperation among units that need to work together”	30.1	75.0
“Units do not coordinate well with each other”	24.7	73.6
“It is often unpleasant to work with staff from other units”	45.2	88.2
“Things ‘fall between the cracks’ when transferring patients from one unit to another”	15.1	73.5
“Important patient care information is often lost during shift changes”	41.1	73.6
“Problems often occur in the exchange of information across units”	34.3	73.6

## Discussion

Patient safety culture, particularly in obstetrics and gynecology, has become a strategic area for improvement to promote the quality of care and patient safety within these units [14, 15]. This study was conducted to evaluate the effectiveness of an education intervention on the quality and safety of care in improving PSC among healthcare professionals working in the obstetrics and gynecology department of Farhat Hached university hospital of Sousse, Tunisia.

In our study, the dimension “Frequency of events reported” (D2) initially had a score of 30.1% and was therefore a dimension to be improved. This score was close to those reported by two other Tunisian studies conducted in intensive care units (ICUs) [2] and in operating rooms [18] and which respectively found scores of 20.8% and 25.6%. Also, a study carried out in a maternity hospital in Switzerland in 2022 showed that this dimension was among the least developed ones with a score of 20.8% [15]. The low score related to reporting AEs in our study would probably be linked to the fear of being judged and blamed for committing an error. Indeed, the reporting of these AEs, whose main goal should be to identify their underlying causes and prevent their future recurrence [19] often results in blame and punishment; which is a main contributor to decreased quality of care and institutional stagnation [20–22]. The dimension “Non-punitive response to error” (D7) may confirm this where, before the intervention, it had a score of 21.1%, meaning that the majority of staff feel that mistakes are blamed on them and that when an event is reported, it is the person who is singled out and not the problem. Others point out that the existing reporting system lacks responsiveness and does not go further than reporting, which explains the low rate of AEs reporting [2, 18].

This low tendency to report and this limited reactivity will in turn limit learning from errors, since AEs represent opportunities for learning, communication and exchange of experiences between caregivers. The dimension “Organizational learning and continuous improvement” (D4) indeed had a very low score during baseline assessment (37.5%), which contrasts with several scores reported in the literature; it was, for example, the most developed dimension in Saudi Arabia [23] and in Latin America [24]. This shows that in these countries, healthcare professionals manage to learn from their mistakes and use them to improve continuously. The low score for this dimension indicates a lack of systems that assist healthcare workers in improving their practice by learning from their mistakes, which makes promoting patient safety and healthcare more challenging [13]. In fact, the use of the learning strategy in healthcare institutions intends to enhance the quality of clinical practice, productivity, lifelong learning, and patient safety [25]. Therefore, mistakes must be regarded as valuable teaching tools and precious opportunities to think, learn, and adjust practices [26]. The presence of a reporting system that coordinates the activity of reporting AEs in a manner that employees can learn from them is one of the best resources and a requirement for enhancing the learning culture within a unit [1]. It is important to note that reporting alone is not sufficient to instore a learning culture. Reporting should be an integral part of a whole chain where reports must be analyzed to know their root causes and undertake corrective actions so that the errors do not happen again [27].

In our intervention, these elements were taken into consideration insofar as awareness was raised to inform about the reporting of AEs and its importance in the improving patient safety and quality of care. Additionally, our study took into account a number of barriers to reporting that were identified in the literature. For example, according to studies on the obstacles to AEs reporting, not providing workers with information about the reporting system, such as what to report, what happens after reporting, who is responsible for reporting, etc., is a significant barrier to reporting [28–30]. The various workshops carried out focused particularly in those technical aspects and on the systemic approach to the occurrence of AEs. The identification of errors in the error room and then the practice of reporting and analyzing the event according to the ALARM method, leading to the identification of systemic root causes seems to allow participants to grasp and anchor the importance of reporting and to change punitive perspective to error. All the more so, whether during face-to-face training or in the self-learning document, the importance of feedback, whether to encourage reporting or to learn from mistakes made was stressed. Indeed, the feedback not only lets the reporter know that his report has been taken into consideration, which is crucial to promote reporting, but it also lets the other professionals know about the error and the circumstances surrounding its occurrence, so they can prevent its renewal [12].

Additionally, during the intervention, concrete examples of improvement actions that were made following AEs occurrence were presented, including training sessions and AEs prevention policies in response to reports received. Mentioning to participants that these improvements were implemented in response to reported AEs makes them more confident that reports will really result in positive changes, which in turn motivates them to report more, reinforces learning culture and strengthens the mindset of continuous improvement where errors and AEs are viewed as learning opportunities [19]. This could explain the significant improvement in the score of the dimensions “organizational learning and continuous improvement” (D4), and “non-punitive response to error” (D7) which could in turn explain the improvement in the dimension “frequency of events reported” (D2).

The score attributed to the dimension “communication openness” (D6) also reflects an unfavorable environment for expression, which could influence the reporting of AEs. Indeed, in our baseline assessment, communication openness had a low score (40.6%) with only 47.9% of participants confirmed that they will not hesitate to speak out freely if they observe something that can adversely affect patients. In healthcare setting, speaking up for patient safety can be defined as assertive, change-oriented, and fluid communication in clinical situations using questions or comments with information, worries, or opinions about safety-related issues [31]. According to prior research, it’s crucial to raise concerns about patient safety in order to prevent medical errors and patient harm [32, 33]. Also, only few participants of baseline assessment (23.3%) affirmed that they are willing to question the decisions or actions of coworkers with more authority and 40.3% of them were neutral or indeed afraid to ask questions when something does not seem right. It can be fear of the reaction of their colleagues and their superiors who can consider the opinion of another professional as a judgment or a devaluation of their skills and can even lead to conflicts between professionals. According to Jason et al’s study [34] that explored barriers to speaking up about patient safety issues, speaking up was made more difficult because of the perception that managers ignored complaints and raised concerns, did not consider safety issues as a priority, or punished employees who voiced their concerns. The fear that coworkers might react unfavorably to someone voicing out or disagree that the situation was truly unsafe was also one of the obstacles to healthcare professionals’ speaking up [34]. Some professionals were unsure about their ability to determine whether a situation is really risky for patients and were even afraid of being fired [34].

The significant increase in this dimension goes hand in hand with Aouicha et al’s study findings [35] that showed that enrolling in patient safety training is associated with a significant increase in communication openness. In our study, healthcare professionals were told and reminded during patient safety trainings that they should speak up if they notice anything harmful. This could explain the improvement in the score of the dimension relating communication openness that significantly increased from 40.6 to 70.6% *p* < 0.001). Also, the fact that the department head-chief was involved in professionals training may play a role in improving communication openness. Indeed, Levine et al. highlighted the importance of managers and supervisors in creating the communication culture that will allow staff to speak up and be heard [36].

In the other hand, teamwork is considered a key element to ensure an optimal care service in hospitals [37]. Indeed, it has been demonstrated that inter-professional teamwork has a positive impact for both employees and patients as it may improve staff satisfaction and well-being as well as it may lead to decreased length of stays, mortality rates, and mediation errors [37–39]. Despite this relevance, teamwork level in healthcare is still unsatisfactory and requires improvement efforts to provide safe patient care [37, 40]. The dynamic nature of obstetrics and gynecology care, the associated dangers, and the need for ongoing adaptation from healthcare providers unavoidably led to an increase in interest in enhancing teamwork in these settings [41]. Thus, teamwork is always being urged to be improved in gynecology and obstetrics departments in order to enhance the safety and quality of maternity care, and prevent mother and infant fatalities [42].

In our study, even though the dimension “Teamwork within units” (D5) was the most developed dimension, its score was not satisfactory (58.2%). Similarly, the dimension related to teamwork across units (D10) had to be improved (score of 31.4%). After the intervention, a significant increase in the score of D5 “teamwork within units” (from 58.2% in pre-test to 79.7% in post-test, *p* < 0.01) and across units (from 31.4 to 76.2% in post-test, *p* < 0.001) was observed. For a matter of fact, a study that aimed to examine the level of teamwork and its relationships with clinical error reporting among Korean nurses showed a significant positive association between error reporting and teamwork [43]. Furthermore, the significant improvement in the dimensions “Communication openness” (D6) and “Non-punitive response to error” (D7) may also explain the significant improvement in “Teamwork within units” (D5). Indeed, studies showed the positive correlation between communication openness and non-punitive environment and teamwork [34, 44]. In their study “The Components of Non-Punitive Environment in Nursing”, Sepp & Tint reported the relationship between teamwork and non-punitive and blame-free environment [44]. Jason et al’s study showed also that PSC and teamwork scores were significantly more positive among professionals indicating they would always speak up than among those who provided reasons for not speaking up [34].

Furthermore, the diversity of professions within healthcare settings underscores the critical need for promoting teamwork, collaborative practice, and Inter-Professional Education (IPE) as essential components of enhancing PSC. In our study, while we focused on healthcare professionals directly involved in gynecology and obstetrics care, it is imperative to recognize the interdisciplinary nature of healthcare delivery. Including various support departments such as management, administration, pharmacists, laboratory technicians, and other healthcare categories like cleaners and drivers fosters a holistic approach to patient care and leverage collective strengths to address complex patient needs more effectively. Integrating IPE initiatives among healthcare workers from different disciplines enables them to learn together, fostering a deeper understanding of each other’s roles, responsibilities, and contributions to better patient outcomes and cultivating a culture of open communication, mutual respect, and shared responsibility [45].

On the other hand, the absence of improvement in “patient safety level” despite enhancements in eight out of ten dimensions of PSC requires a nuanced examination. Firstly, it’s important to distinguish between perceptions of patient safety culture and tangible changes in patient safety outcomes. While improvements in dimensions such as teamwork and communication openness may positively influence healthcare professionals’ perceptions of safety culture, these changes may not immediately translate into measurable improvements in patient safety outcomes, such as reductions in AEs. Furthermore, patient safety is a multifaceted construct influenced by various factors beyond organizational culture, including systemic issues, individual behaviors, and external pressures. Improving patient safety requires addressing these complex factors through evidence-based practices, system-level interventions, and a commitment to continuous learning and improvement. As a result, improvements in PSC dimensions alone may not fully capture the complexity of patient safety challenges and may require complementary strategies to effect meaningful change.

Similarly, the observed improvement in the dimension of “frequency of adverse events reported” and “non-punitive response to error” suggests that there was indeed a positive shift in healthcare professionals’ attitudes towards adverse event reporting and error management. These dimensions primarily reflect perceptions, attitudes, and organizational culture surrounding reporting practices rather than the actual frequency of reporting or the implementation of reporting behaviors. The outcome dimension “number of adverse events reported” represents a more tangible measure of actual reporting behavior and practice. It reflects the concrete actions taken by healthcare professionals to report AEs, rather than their perceptions or attitudes towards reporting.

The discrepancy between the improvements in attitude-related dimensions and the lack of change in reporting behavior may indeed be attributed to the distinction between attitudes and practices. While improvements in attitudes and organizational culture are important precursors to behavior change, they may not always directly translate into observable changes in practice, especially in complex healthcare environments with various systemic and practical barriers to reporting.

### Study limitations

Our study encountered some limitations, which are crucial to acknowledge. Firstly, reliance on self-reported data for assessing PSC introduces the possibility of social desirability bias and response bias, potentially influencing the accuracy and reliability of reported perceptions. Despite efforts to mitigate these biases through the utilization of validated assessment tool, and the insurance the anonymity and confidentiality, the subjective nature of self-reported data remains a limitation of our study. Furthermore, the quasi-experimental design utilized in our study, while practical for real-world settings, presents inherent limitations. The absence of a control group and randomization may limit our ability to establish causal relationship. Additionally, the relatively small sample size and the inclusion of a department of a single hospital may impact the generalizability of our findings to broader populations or healthcare settings. Lastly, the absence of the evaluation of the training program limited the identification of its strengths and weaknesses, hindering guidance for future improvements and diminishing tangible evidence of the perceived training effectiveness by participants.

### Study implications

Our study provides valuable insights into the effectiveness of educational interventions in improving PSC within gynecology-obstetrics departments, and several implications for research, practice, and policy emerge from its finding. From a policy standpoint, policymakers should recognize the importance of investing in educational initiatives and creating supportive environments for patient safety within healthcare settings. In addition, there is a clear need for policies that promote the adoption of evidence-based practices and facilitate collaboration among healthcare professionals to enhance PSC. Policymakers should also consider allocating resources for the development and implementation of educational interventions and quality improvement initiatives aiming at improving patient safety outcomes. In terms of practice, healthcare institutions can leverage our findings to develop tailored interventions aiming at improving PSC and enhancing teamwork and communication within clinical settings. Practitioners should recognize the value of ongoing education and training programs in fostering a culture of safety among healthcare professionals. These trainings should include not only department staff but also the other supporting departments and the different healthcare categories. From a research perspective, future studies should prioritize the implementation of robust evaluation mechanisms to assess the effectiveness of training programs comprehensively. This may involve incorporating objective outcome measures, such as observed changes in clinical practices or patient outcomes. Additionally, researchers should explore alternative study designs, such as randomized controlled trials, to strengthen causal inferences.

## Conclusions

PSC is an important determinant of patient safety in healthcare settings as it influences patient safety outcomes. Improving the PSC remains a big challenge and takes time to ensure long-lasting results.

In our study, although most of the PSC dimensions have improved after the training program, others require an action plan. The sustainability of the improvement noted requires the intensification of patient safety management programs. Future research should focus on the implementation and evaluation of participatory institutional risk management programs prioritizing the PSC development. The sustainability of a PSC depends on the collaboration of healthcare workers from different disciplines. Staff commitment at all levels remains the cornerstone of any continuous improvement in the area of patient safety.

## Data Availability

On demand, from corresponding author.

## Abbreviations

AE(s): Adverse Event(s)
APR: Average Positive Response
HSOPSC: Hospital Survey On Patient Safety Culture
IPE: Inter-Professional Education
PSC: Patient Safety Culture

## References

[1] Tlili MA, Aouicha W, Gambashidze N, Ben Cheikh A, Sahli J, Weigl M (2024). A retrospective analysis of adverse events reported by Tunisian intensive care units’ professionals. BMC Health Serv Res.

[2] Tlili MA, Aouicha W, Sahli J, Ben Cheikh A, Mtiraoui A, Ajmi T (2022). Assessing patient safety culture in 15 intensive care units: a mixed-methods study. BMC Health Serv Res.

[3] Aouicha W, Tlili MA, Limam M, Snéne M, Ben Dhiab M, Chelbi S (2021). Evaluation of the Impact of Intraoperative Distractions on Teamwork, stress, and workload. J Surg Res.

[4] Hüner B, Derksen C, Schmiedhofer M, Lippke S, Riedmüller S, Janni W (2023). Reducing preventable adverse events in obstetrics by improving interprofessional communication skills – results of an intervention study. BMC Pregnancy Childbirth.

[5] Skoogh A, Hall-Lord ML, Bååth C, Bojö A-KS (2021). Adverse events in women giving birth in a labor ward: a retrospective record review study. BMC Health Serv Res.

[6] Lippke S, Wienert J, Keller FM, Derksen C, Welp A, Kötting L (2019). Communication and patient safety in gynecology and obstetrics - study protocol of an intervention study. BMC Health Serv Res.

[7] Tanaka K, Eriksson L, Asher R, Obermair A (2019). Incidence of adverse events, preventability and mortality in gynaecological hospital admissions: a systematic review and meta-analysis. Aust N Z J Obstet Gynaecol.

[8] Ghali H, Cheikh AB, Bhiri S, Fredj SB, Layouni S, Khefacha S et al. Évènements indésirables dans un hôpital universitaire tunisien: incidence et facteurs de risque. Santé Publique 2020;Vol 32:189–98. 10.3917/spub.202.0189. 10.3917/spub.202.0189 35724212

[9] Letaief M, El Mhamdi S, Siddiqi S, Letaief R, Morjane A, Hamdi A (2020). A prospective Assessment of adverse events in 3 Digestive surgery Departments from Central Tunisia. J Patient Saf.

[10] Bouafia N, Bougmiza I, Bahri F, Letaief M, Astagneau P, Njah M. Ampleur Et impact des évènements indésirables graves liés aux soins: étude d’incidence dans Un hôpital Du Centre-Est tunisien. Pan Afr Med J. 2013;16. 10.11604/pamj.2013.16.68.1161. 10.11604/pamj.2013.16.68.1161 PMC3976656 24711868

[11] Hafezi A, Babaii A, Aghaie B, Abbasinia M (2022). The relationship between patient safety culture and patient safety competency with adverse events: a multicenter cross-sectional study. BMC Nurs.

[12] Kilcullen MP, Bisbey TM, Ottosen MJ, Tsao K, Salas E, Thomas EJ (2022). The Safer Culture Framework: an application to Healthcare based on a Multi-industry Review of Safety Culture Literature. Hum Factors J Hum Factors Ergon Soc.

[13] Mistri IU, Badge A, Shahu S (2023). Enhancing patient Safety Culture in hospitals. Cureus.

[14] Ansari SP, Rayfield ME, Wallis VA, Jardine JE, Morris EP, Prosser-Snelling E (2020). A safety evaluation of the impact of maternity-orientated human factors training on Safety Culture in a Tertiary maternity unit. J Patient Saf.

[15] Staines A, Lécureux E, Rubin P, Baralon C, Farin A (2020). Impact of TeamSTEPPS on patient safety culture in a Swiss maternity ward. Int J Qual Health Care.

[16] Occelli P, Quenon J-L, Kret M, Domecq S, Delaperche F, Claverie O (2013). Validation of the French version of the Hospital Survey on Patient Safety Culture questionnaire. Int J Qual Health Care J Int Soc Qual Health Care ISQua.

[17] Occelli P, Quenon J-L, Djihoud A. Mesure de la culture de sécurité des soins en milieu hospitalier - Guide d’utilisation de l’outil de mesure 2010.

[18] Aouicha W, Tlili MA, Sahli J, Mtiraoui A, Ajmi T, Said Latiri H (2022). Patient safety culture as perceived by operating room professionals: a mixed-methods study. BMC Health Serv Res.

[19] Tlili MA, Aouicha W, Sahli J, Mtiraoui A, Ajmi T, Laatiri H (2022). An intervention to optimize attitudes toward adverse events reporting among Tunisian critical care nurses. J Patient Saf.

[20] Brborović O, Brborović H, Nola IA, Milošević M (2019). Culture of Blame—An ongoing burden for doctors and patient safety. Int J Environ Res Public Health.

[21] Kaur AP, Levinson AT, Monteiro JFG, Carino GP (2019). The impact of errors on healthcare professionals in the critical care setting. J Crit Care.

[22] Unoki T, Hamamoto M, Sakuramoto H, Shirasaka M, Moriyasu M, Zeng H, et al. Influence of mutual support and a culture of blame among staff in acute care units on the frequency of physical restraint use in patients undergoing mechanical ventilation. Acute Med Surg. 2020;7. 10.1002/ams2.479. 10.1002/ams2.479 PMC6971454 31988791

[23] Alrowely Z, Ghazi Baker O. Assessing Building blocks for Patient Safety Culture—a quantitative Assessment of Saudi Arabia. Risk Manag Healthc Policy 2019;Volume 12:275–85. 10.2147/RMHP.S223097. 10.2147/RMHP.S223097 PMC6902844 31827340

[24] Camacho-Rodríguez DE, Carrasquilla-Baza DA, Dominguez-Cancino KA, Palmieri PA (2022). Patient Safety Culture in Latin American hospitals: a systematic review with Meta-analysis. Int J Environ Res Public Health.

[25] Goula A, Stamouli M-A, Latsou D, Gkioka V, Kyriakidou N (2021). Learning Organizational Culture in Greek Public hospitals. Int J Environ Res Public Health.

[26] Ferretti E, Rohde K, Moore GP, Daboval T (2019). Catch the moment: the power of turning mistakes into ‘precious’ learning opportunities. Paediatr Child Health.

[27] Singh G, Patel RH, Boster J (2022). Root cause Analysis and Medical Error Prevention.

[28] Woo MWJ, Avery MJ (2021). Nurses’ experiences in voluntary error reporting: an integrative literature review. Int J Nurs Sci.

[29] Aljabari S, Kadhim Z (2021). Common barriers to reporting medical errors. Sci World J.

[30] Afaya A, Konlan KD, Kim Do H (2021). Improving patient safety through identifying barriers to reporting medication administration errors among nurses: an integrative review. BMC Health Serv Res.

[31] Lee SE, Choi J, Lee H, Sang S, Lee H, Hong HC. Factors influencing nurses’ willingness to speak up regarding Patient Safety in East Asia: a systematic review. Risk Manag Healthc Policy 2021;Volume 14:1053–63. 10.2147/RMHP.S297349. 10.2147/RMHP.S297349 PMC7966392 33737846

[32] Hémon B, Michinov E, Guy D, Mancheron P, Scipion A (2020). Speaking up about errors in routine clinical practice: a Simulation-based intervention with nursing students. Clin Simul Nurs.

[33] Szymczak JE (2016). Infections and interaction rituals in the organisation: clinician accounts of speaking up or remaining silent in the face of threats to patient safety. Sociol Health Illn.

[34] Etchegaray JM, Ottosen MJ, Dancsak T, Thomas EJ (2020). Barriers to speaking up about patient safety concerns. J Patient Saf.

[35] Aouicha W, Tlili MA, Sahli J, Dhiab MB, Chelbi S, Mtiraoui A (2021). Exploring patient safety culture in emergency departments: a Tunisian perspective. Int Emerg Nurs.

[36] Levine KJ, Carmody M, Silk KJ (2020). The influence of organizational culture, climate and commitment on speaking up about medical errors. J Nurs Manag.

[37] Dinius J, Philipp R, Ernstmann N, Heier L, Göritz AS, Pfisterer-Heise S (2020). Inter-professional teamwork and its association with patient safety in German hospitals—A cross sectional study. PLoS ONE.

[38] Liu J, Masiello I, Ponzer S, Farrokhnia N (2019). Interprofessional teamwork versus fast track streaming in an emergency department—An observational cohort study of two strategies for enhancing the throughput of orthopedic patients presenting limb injuries or back pain. PLoS ONE.

[39] Kang XL, Brom HM, Lasater KB, McHugh MD (2020). The Association of nurse–physician Teamwork and Mortality in Surgical patients. West J Nurs Res.

[40] Rosen MA, DiazGranados D, Dietz AS, Benishek LE, Thompson D, Pronovost PJ (2018). Teamwork in healthcare: key discoveries enabling safer, high-quality care. Am Psychol.

[41] Neuhaus C, Lutnæs DE, Bergström J (2022). Emergence of power and complexity in obstetric teamwork. PLoS ONE.

[42] Harris J, Beck S, Ayers N, Bick D, Lamb BW, Aref-Adib M (2022). Improving teamwork in maternity services: a rapid review of interventions. Midwifery.

[43] Hwang J-I, Ahn J (2015). Teamwork and clinical error reporting among nurses in Korean hospitals. Asian Nurs Res.

[44] Sepp J, Tint P. The components of non-punitive environment in nursing. Saf Technog Environ. 2017;8. 10.1515/ste-2017-0004.

[45] Alsabri M, Boudi Z, Lauque D, Dias RD, Whelan JS, Östlundh L (2022). Impact of Teamwork and Communication Training interventions on Safety Culture and Patient Safety in Emergency departments: a systematic review. J Patient Saf.

